# Emergence and Persistence of High-Risk Clones Among MDR and XDR *A. baumannii* at a Brazilian Teaching Hospital

**DOI:** 10.3389/fmicb.2018.02898

**Published:** 2019-01-04

**Authors:** Laís Calissi Brisolla Tavares, Francielli Mahnic de Vasconcellos, William Vaz de Sousa, Taisa Trevizani Rocchetti, Alessandro Lia Mondelli, Adriano Martison Ferreira, Augusto Cezar Montelli, Terue Sadatsune, Monique Ribeiro Tiba-Casas, Carlos Henrique Camargo

**Affiliations:** ^1^Faculdade de Medicina da Universidade de São Paulo, São Paulo, Brazil; ^2^Centro de Bacteriologia, Instituto Adolfo Lutz, São Paulo, Brazil; ^3^Faculdade de Medicina de Botucatu, Botucatu, Brazil

**Keywords:** *Acinetobacter baumannii*, oxacillinases, resistance epidemiology, healthcare associated infections, clonal complexes, MLST

## Abstract

Dissemination of carbapenem-resistant *Acinetobacter baumannii* is currently one of the priority themes discussed around the world, including in Brazil, where this pathogen is considered endemic. A total of 107 carbapenem-resistant *A. baumannii* (CRAB) isolates were collected from patients with bacteraemia attended at a teaching hospital in Brazil from 2008 to 2014. From these samples, 104 (97.2%) carried *bla*_OXA−23−like_, all of them associated with IS*Aba1* The *bla*_OXA−231_ (1.9%) and *bla*_OXA−72_ (0.9%) genes were also detected in low frequencies. All isolates were susceptible to minocycline, and 38.3% of isolates presented intermediate susceptibility to tigecycline (MIC = 4 μg/ml). Molecular typing assessed by multi-locus sequence typing demonstrated that the strains were mainly associated with clonal complexes CC79 (47.4%), followed by CC1 (16.9%), and CC317 (18.6%), belonging to different pulsotypes and in different prevalences over the years. Changes in the clones' prevalence reinforce the need of identifying and controlling CRAB in hospital settings to preserve the already scarce therapeutic options available.

## Introduction

Emergence and dissemination of carbapenem-resistant *Acinetobacter baumannii* (CRAB) is currently one of the priority themes discussed around the world (Higgins et al., 2010; US Centers for Disease Control Prevention. *Antibiotic Resistance Threats in the United States*., 2013; World Health Organization, 2017). In Brazil, a continental country in which healthcare associated infections rates are distinctly high (Fortaleza et al., 2017), CRAB is considered endemic (Rossi, 2011) and *Acinetobacter* infections present the highest mortality rates among ICU patients with bacterial bloodstream infections (Marra et al., 2011). Carbapenem resistance in *Acinetobacter* is usually mediated by carbapenem-hydrolysing class D β-lactamase (CHDL), mainly codified by the *bla*_OXA−23_-like, *bla*_OXA−24_-like, *bla*_OXA−58_-like and *bla*_OXA−143_-like genes (Zarrillii et al., 2013).

Molecular epidemiology of CRAB highlights the prevalence of International Clone 1 (Clonal Complex CC1) worldwide, while International Clones 2 (CC2) and 3 (CC3) are prevalent in Europe and in the United States (Zarrilli et al., 2009; Karah et al., 2012; Zarrillii et al., 2013). In Brazil and other Latin American countries (Clímaco et al., 2013; Medeiros and Lincopan, 2013; Stietz et al., 2013; Rodríguez et al., 2016; Escandón-Vargas et al., 2017), clonal complexes CC1, CC15 are predominant, along with CC79, which has also been identified in Spain and in the United States (Villalón et al., 2011; Mosqueda et al., 2014; Kanamori et al., 2016).

Circulation of a limited number of lineages of multidrug- (MDR) and extensively-drug resistance (XDR) *A. baumannii* underscores the need for surveillance and effective implementation of measures to contain their dissemination.

To determine the occurrence of high-risk clones CRAB circulating in a Brazilian hospital, we evaluated their antimicrobial susceptibility and clonality in isolates recovered from bloodstream infections in patients attended at a teaching hospital in inner Brazil.

## Materials and methods

### Epidemiological design

This was an observational retrospective study performed with 107 carbapenem-resistant *A. baumannii* isolates recovered from not-repeated patients with bacteraemia attending at Botucatu Medical School Hospital/UNESP (BMSH/UNESP), from 2008 to 2014. The study was approved as a retrospective study by the Local Research Ethics Committee (Process CAAE 49985115.5.0000.0059). We were granted an exemption from the requirement to obtain written informed-consent from the participants and/or their legal guardians because the isolates included in the study had already been stored, on an ongoing basis, in the Culture Collection of the Department of Microbiology and Immunology, UNESP, Botucatu, São Paulo, Brazil.

### Settings and *A. baumannii* isolates

BMSH/UNESP is a 415-bed (52 intensive care unit beds) tertiary regional reference hospital, located in the inner of the State of Sao Paulo, Brazil. For this study, frozen isolates stocked in deep-freezer were recovered in Brain-Heart Infusion (BHI) broth and streaked onto BHI Agar plates. *Acinetobacter* isolates were initially identified by morphological and biochemical characteristics (Gram stain, oxidase-negative, catalase-positive, glucose oxidation, ability to grow at 42° and 44°C) (Vaneechoutte et al., 2015). *A. baumannii* species was screened by PCR detection of *bla*_OXA−51−like_ (Woodford et al., 2006) and *glt*A genes (Wong et al., 2014). A subset of randomly selected isolates was submitted to ITS and/or *rpo*B gene sequencing (Chang et al., 2005; La Scola et al., 2006). As MLST was carried out for each pulsotype (see below), *A. baumannii* identification was also confirmed by this method.

### Detection of oxacillinase genes and IS*Aba1*

PCR for CHDL oxacillinases-encoding genes (*bla*_OXA−23−like_, *bla*_OXA−24/40−like_, *bla*_OXA−143−like_ and *bla*_OXA−58−like_) and other carbapenemases (*bla*_KPC_, *bla*_NDM_, *bla*_SPM_, *bla*_IMP_, *bla*_VIM_, *bla*_OXA−48_) was performed in all isolates as previously described (Woodford et al., 2006; Higgins et al., 2010; Poirel et al., 2011). Full sequencing of the *bla*_OXA−24_-like and the *bla*_OXA−143−like_ was carried out for allele determination (Héritier et al., 2005; Higgins et al., 2009; Cayô et al., 2014). Presence of the IS*Aba1* upstream the *bla*_OXA_ genes was also investigated by PCR mapping, employing the IS*Aba1* forward primer (Segal et al., 2005) and the *bla*_OXA_ reverse primers (Woodford et al., 2006).

### Antimicrobial susceptibility testing

Minimum inhibitory concentration (MIC) values were determined for ampicillin-sulbactam, minocycline, and tetracycline (broth microdilution method), imipenem, meropenem, tigecycline, and polymyxin B (E-test, BioMeriéux, Marci l'Etoile, France). The susceptibility profile of the isolates was completed by disc-diffusion method to amikacin, cefepime, ceftazidime, cefotaxime, ciprofloxacin, gentamicin, levofloxacin, piperacillin-tazobactam, trimethoprim-sulfamethoxazole, ticarcillin-clavulanate, and tobramycin. Breakpoints employed to define susceptibility, intermediate or resistance followed CLSI recommendations (Clinical Laboratory Standards Institute, 2015), except for tigecycline, for which the US Food and Drug Administration breakpoints were applied (susceptible: ≤ 2 μg ml^−1^; resistant: ≥8 μg ml−1). Isolates were categorized as multidrug- (MDR, non-susceptibility to ≥1 agent in ≥3 antimicrobial categories among aminoglycosides, antipseudomonal carbapenems, antipseudomonal fluoroquinolones, antipseudomonal penicillins + β-lactamase inhibitors, extended-spectrum cephalosporins, folate pathway inhibitors, penicillins + β-lactamase inhibitors, polymyxins, tetracyclines) or extensively-drug-resistant (XDR, non-susceptibility to ≥1 agent in all but ≤ 2 categories, as described earlier) (Magiorakos et al., 2012).

### Determination of the electrophoretic pattern by PFGE

Genetic diversity among all the 107 *A. baumannii* isolates were investigated by PFGE (Seifert et al., 2005). Macrorestriction was performed with ApaI (Promega) and DNA digested fragments were resolved using a CHEF-DR-III (Bio-Rad). Dendrogram was generated with BioNumerics v.7.6.2 (Applied Maths, Sint-Martens-Latem, Belgium) based on the Dice similarity using the UPGMA method, with tolerance and optimization parameters set at 1.5%. Clusters were defined as isolates with similarity ≥87% and named with capital letters (A to K) while pulsotypes were defined as each electrophoretic pattern with 100% similarity (named with capital letters and numbers, from A1 to K4).

### MLST analysis

MLST was performed in a representative isolate of each pulsotype as per the Institute Pasteur protocol (https://pubmlst.org/abaumannii/info/primers_Pasteur.shtml), in order to sequence the internal region of the genes *gltA, fusA, recA, cpn60, pyrG, rplB* and *rpoB*. PCR products were purified with enzyme ExoSAP-IT (Affymetrix) according to manufacturer's instructions. Nucleotide sequences were obtained using an Applied 3730 Automatic Sequencer (Applied Biosystems). The results were analyzed on BioNumerics v.7.6.2 (Applied Maths, Sint-Martens-Latem, Belgium), and compared with the Institute Pasteur database (https://pubmlst.org/abaumannii/); clonal complexes were determined by the eBurst algorithm (http://eburst.mlst.net/).

## Results

### *A. baumannii* isolates and detection of oxacillinase genes

The 107 isolates were recovered from non-repetitive patients with bacteremia attending a teaching hospital in the State of São Paulo, Brazil, between 2008 and 2014. These isolates were recovered from blood (96.3%) or vascular catheter (3.7%).

All isolates were confirmed as *A. baumannii* species by PCR detection of *bla*_OXA−51−like_ and/or sequencing of the *rpo*B and ITS genes, as well as by the MLST analysis. Out of these isolates, 104 (97.2%) carried the *bla*_OXA−23−like_ with IS*Aba1* upstream; remaining strains carried *bla*_OXA−231_ (*bla*_OXA−143−like_; *n* = 2; 1.9%) or *bla*_OXA−72_ (*bla*_OXA−24−like_; *n* = 1; 0.9%). The *bla*_OXA−58−like_ gene was not detected, as well as the additional carbapenemases investigated.

### Antimicrobial susceptibility testing

According to the susceptibility test, 39.3% of isolates were considered MDR, and 60.7% XDR. The entire population evaluated confirmed resistance to imipenem, meropenem, ciprofloxacin, piperacillin-tazobactam and levofloxacin, while susceptibility to minocycline was observed in all the isolates. Resistance rates to other antimicrobials tested by disc-diffusion method were: amikacin (89.7%), cefepime (98.1%), ceftazidime (96.2%), cefotaxime (99.0%), gentamicin (76.6%), trimethoprim-sulfamethoxazole (68.2%), ticarcillin-clavulanate (99.0%), and tobramycin (68.2%). The MIC_50_/MIC_90_ (μg/ml) values were calculated for each antimicrobial agent, as follows: imipenem (>32/>32), meropenem (>32/>32), tetracycline (8/16), ampicillin-sulbactam (16:8/32:16), minocycline (0.25/0.5) and tigecycline (2/3). Although resistance to tigecycline was not detected, 38.3% of isolates presented intermediate susceptibility to this drug (according to FDA breakpoints).

Distribution of susceptibility rates among the clones (Table 1) evidenced high-rates of non-susceptibility to cephalosporins, carbapenems, quinolones and ticarcillin-clavulanic acid. For the aminoglycosides, the highest susceptibility rate to amikacin was detected for isolates belonging to CC1 (54.5%), while gentamycin and tobramycin were more active against isolates belonging to CC317 (60.7% and 53.6% of susceptibility, respectively). Furthermore, isolates belonging to CC317 presented the highest susceptibility rates to sulfamethoxazole-trimethoprim (92.9%), tigecycline (75%), and ampicillin-sulbactam (53.6%).

**Table 1:** Distribution (frequency and %) of antimicrobial susceptibility (S) and non-susceptibility (NS, intermediate + resistance) among the clones of *A. baumannii*.

**Antimicrobial1**	**CC1 (*****n*** = **11)**				**CC15 (*****n*** = **10)**				**CC79 (*****n*** = **53)**				**ST317 (*****n*** = **28)**				**Others (ST25, ST107, ST22) (*****n*** = **5)**			
	**S**	**(%)**	**NS**	**(%)**	**S**	**(%)**	**NS**	**(%)**	**S**	**(%)**	**NS**	**(%)**	**S**	**(%)**	**NS**	**(%)**	**S**	**(%)**	**NS**	**(%)**
GN	0	(0)	11	(100)	0	(0)	10	(100)	5	(9.4)	48	(90.6)	17	(60.7)	11	(39.3)	3	(60)	2	(40)
TOB	0	(0)	11	(100)	1	(10)	9	(90)	14	(26.4)	39	(73.6)	15	(53.6)	13	(46.4)	4	(80)	1	(20)
AK	6	(54.5)	5	(45.5)	0	(0)	10	(100)	2	(3.8)	51	(96.2)	0	(0)	28	(100)	2	(40)	3	(60)
IMP	0	(0)	11	(100)	0	(0)	10	(100)	0	(0)	53	(100)	0	(0)	28	(100)	0	(0)	5	(100)
MEM	0	(0)	11	(100)	0	(0)	10	(100)	0	(0)	53	(100)	0	(0)	28	(100)	0	(0)	5	(100)
CIP	0	(0)	11	(100)	0	(0)	10	(100)	0	(0)	53	(100)	0	(0)	28	(100)	0	(0)	5	(100)
LEV	0	(0)	11	(100)	0	(0)	10	(100)	0	(0)	53	(100)	0	(0)	28	(100)	0	(0)	5	(100)
PTZ	0	(0)	11	(100)	0	(0)	10	(100)	0	(0)	53	(100)	0	(0)	28	(100)	0	(0)	5	(100)
TIM	0	(0)	11	(100)	0	(0)	10	(100)	0	(0)	53	(100)	1	(3.6)	27	(96.4)	0	(0)	5	(100)
CTX	0	(0)	11	(100)	0	(0)	10	(100)	0	(0)	53	(100)	0	(0)	28	(100)	0	(0)	5	(100)
CAZ	0	(0)	11	(100)	0	(0)	10	(100)	0	(0)	53	(100)	0	(0)	28	(100)	0	(0)	5	(100)
FEP	0	(0)	11	(100)	0	(0)	10	(100)	0	(0)	53	(100)	0	(0)	28	(100)	0	(0)	5	(100)
SXT	5	(45.5)	6	(54.5)	1	(10)	9	(90)	2	(3.8)	51	(96.2)	26	(92.9)	2	(7.1)	0	(0)	5	(100)
SAM	0	(0)	11	(100)	2	(20)	8	(80)	2	(3.8)	51	(96.2)	15	(53.6)	13	(46.4)	1	(20)	4	(80)
PB	11	(100)	0	(0)	10	(100)	0	(0)	53	(100)	0	(0)	28	(100)	0	(0)	5	(100)	0	(0)
TE	8	(72.7)	3	(27.3)	2	(20)	8	(80)	18	(34)	35	(66)	21	(75)	7	(25)	2	(40)	3	(60)
MIN	11	(100)	0	(0)	10	(100)	0	(0)	53	(100)	0	(0)	28	(100)	0	(0)	5	(100)	0	(0)
TG	0	(0)	11	(100)	7	(70)	3	(30)	5	(9.4)	48	(90.6)	25	(89.3)	3	(10.7)	3	(60)	2	(40)

*CC, clonal complex; ST, sequence type. 1 GN, gentamycin; TOB, tobramycin; AK, amikacin; IMP, imipenem; MEM, meropenem; CIP, ciprofloxacin; LEV, levofloxacin; PTZ, piperacillin-tazobactam; TIM, ticarcillin-clavulanic acid; CTX, cefotaxime; CAZ; ceftazidime; FEP, cefepime; SXT, sulfamethoxazole-trimethoprim; SAM, ampicillin-sulbactam; PB, polymyxin B; TE, tetracycline; MIN, minocycline; TG, tigecycline*.

### Molecular typing

**Figure 1:**
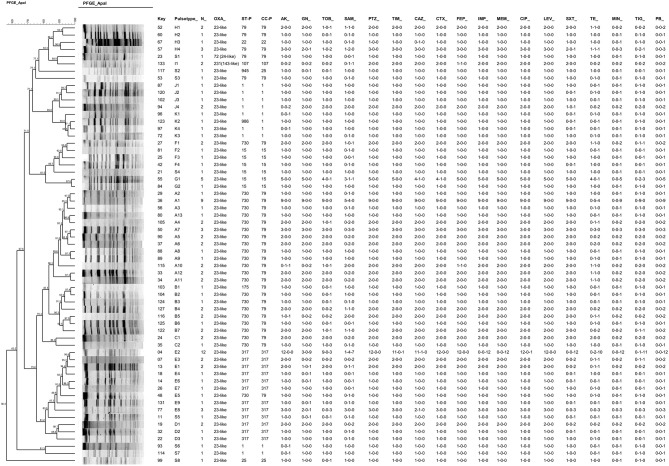
Dendrogram resulted from PFGE analysis of one representative isolate of each pulsotype (*n* = 61) combined with antimicrobial susceptibility results of each pulsotype. Information on additional OXA-enzymes, PFGE cluster, STs and CCs defined by MLST are shown. The susceptibility results are indicated in occurrences for each pulsotype in the following order: resistant-intermediate-susceptible number of strains (i.e., 1-1-0 for a given antimicrobial means 1 resistant strain; 1 intermediate and zero susceptible). AK, amikacin; GN, gentamycin; TOB, tobramycin; SAM, ampicillin-sulbactam; PTZ, piperacillin-tazobactam; TIM, ticarcillin-clavulanic acid; CAZ; ceftazidime; CTX, cefotaxime; FEP, cefepime; IMP, imipenem; MEM, meropenem; CIP, ciprofloxacin; LEV, levofloxacin; SXT, sulfamethoxazole-trimethoprim; TE, tetracycline; MIN, minocycline; TG, tigecycline; PB, polymyxin B. Key: univocal identification number; ST, sequence type; CC, Clonal Complex.

The 107 CRAB isolates were distributed into 53 PFGE pulsotypes belonging to eleven clusters (A, B, C, D, E, F, G, H, I, J, K) and eight additional pulsotypes with a single isolate (Figure 1). By extrapolating the results of MLST to isolates presenting the same pulsotypes, we observed the frequencies of 49.5% of strains belonging to clonal complexes (and sequence types) CC79 (ST79, 8.4%; ST175, 0.9% and ST730, 40.2%); 26.2% for CC317 (ST317, 100%), 10.3% for CC1 (ST1, 9.3%; ST986, 0.9%); 9.3% for CC15 (ST15, 9.3%), 1.9% for CC25 (ST25, 0.9%; ST945, 0.9%), 1.9% for CC107 (ST107, 100%), and 0.9% for CC22 (ST22 0.9%). With the exception of the PFGE cluster I, which presented the *bla*_OXA−231_ allele, the *bla*_OXA−23−like_ gene associated with upstream IS*Aba1* was present in all clusters (Figure 1).

**Figure 2:**
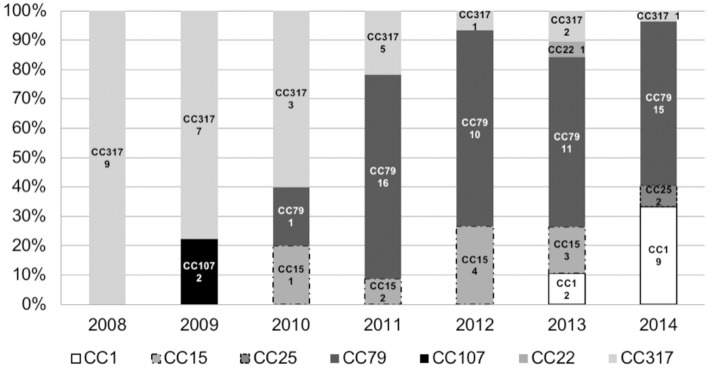
Distribution of Sequence Types (ST) or Clonal Complexes (CC) defined by MLST (Institute Pasteur protocol) over the years. Numbers below the CC or ST indicate number of occurrences (*n* = 107).

Distribution of clones over the time evidenced the emergence of *A. baumannii* pulsotypes belonging to clonal complex 79 in 2010, which became endemic in the institution until 2014 (Figure 2). The CC79 strains (*n* = 53) were distributed into 29 pulsotypes (Figure 1), recovered from 2010 to 2014. Although the pulsotype A1 was the most numerous (9 isolates), it was distributed in 2011 (4 isolates), 2013 (4 isolates) and 2014 (1 isolate). The remaining 28 pulsotypes were represented by 1, 2, or 3 isolates, each, and were recovered from the period comprised between 2010 over 2014. The 2014 isolates (the year with the largest number of isolates (13) belonging to CC79) were represented by 9 different pulsotypes with only 1 or 2 isolates in each electrophoretic pattern.

On the other hand, reduction in the frequency of CC317 was remarkable (Figure 2). The CC317 isolates (*n* = 28) were typed into 12 pulsotypes. A major clone (pulsotype E2) was identified comprising 12 isolates recovered from 2008 (6), 2009 (2), 2010 (2), 2011 (1), 2012 (1). Conversely, the remaining 11 pulsotypes were represented by 1, 2, or 3 isolates, each, recovered over the period of 2008–2013.

CC1 strains were detected in 2013 at frequency lower than 10% (2 isolates) but in 2014 this clonal complex represented more than 30% of the CRAB isolated from BSI (9 isolates belonging to 8 different pulsotypes), same year that CC25 strains were firstly detected.

## Discussion

In this study, we verified the high prevalence of *bla*_OXA−23−like_ in MDR and XDR strains isolated from patients with bacteraemia caused by carbapenem-resistant *Acinetobacter baumannii* in a teaching hospital in Brazil. In addition, changes in the clonal structure of circulating strains was verified, with predominance of Clonal Complexes CC1, CC15, CC79, and CC317.

The oxacillinase genes are differently distributed around the globe, with the *bla*_OXA−23_-like gene being predominant and widespread in several countries (Evans and Amyes, 2014). In this study, the *bla*_OXA−23−like_ gene was identified in most of the isolates belonging to different PFGE restriction patterns and MLST clonal complexes, reinforcing the already known data from this country (Medeiros and Lincopan, 2013). In addition, we also detected the *bla*_OXA−72_ and *bla*_OXA−231_ genes, but in lower frequencies (*n* = 2, 1.9%; and *n* = 1; 0.9%, respectively), consistent with previous reports that detected both genes in this country over the last years (Gionco et al., 2012; Vasconcelos et al., 2015; Camargo et al., 2016; Pagano et al., 2017). Absence of another carbapenemases reinforces the role of oxacillinases among the isolates from Brazil, a country in which CRAB is considered highly prevalent (Rossi, 2011), but we cannot exclude the occurrence of other emergent carbapenemases (such as OXA-235-like or TMB-like), not sought in this study.

Minocycline was active against all the isolates, even if they presented MDR or XDR phenotypes. Although the study performed by Wang and colleagues had demonstrated a higher susceptibility rate to minocycline by microdilution method in CRAB compared to epsilometric method, MIC values identified in the isolates of this study were far below the intermediate value for this drug, confirming their susceptibilities (Wang et al., 2016). This antimicrobial is commonly effective against carbapenem-resistant *Acinetobacter baumannii* (Lashinsky et al., 2017; Poirel et al., 2017), although resistance is already being observed (Cheah et al., 2016; Pournaras et al., 2017; Vasconcellos et al., 2017a). On the other hand, resistance rates to other antimicrobial classes were remarkable. Aminoglycosides, for instance, presented susceptibilities rates ranging from 31.8% to tobramycin to only 9.3% to amikacin. Despite the controversies, tigecycline can be considered one of the few therapeutic options for treatment of MDR infection in skin and soft tissue infections and meningitis (Montravers et al., 2013; Kooli et al., 2016; Lauretti et al., 2017). Still tigecycline is not recommended for the treatment of ventilator-associated pneumonia and BSI (http://www.fda.gov/drugs/drugsafety/ucm224370.htm), we evaluate the tigecycline activity in our BSI isolates with surveillance purposes and no resistance was observed, even this phenotype becomes progressively more common in *A. baumannii* in several countries (Navon-Venezia et al., 2007; Al-Sweih et al., 2011; Montravers et al., 2013; Sun et al., 2013; Provasi Cardoso et al., 2016; Vasconcellos et al., 2017a; Royer et al., 2018).

Among the CRAB isolates from a single hospital, we identified the predominance of CC79 (49.5%), CC1 (10.3%), and CC15 (9.3%), corresponding to 69.2% of all the isolates evaluated over the entire period. This finding is well documented in Brazil as well as the occurrence of isolates belonging to the CC25, which seems to configure an emerging clone in our country (Chagas et al., 2014; Campos et al., 2015; Camargo et al., 2016; Provasi Cardoso et al., 2016; Vasconcellos et al., 2017a,b; Royer et al., 2018), although ST25 has already been detected worldwide (Sahl et al., 2015). ST107 was also identified, corresponding to the two isolates carrying OXA-231, which reinforces the association between ST107 and OXA-231 in Brazil (Camargo et al., 2016; Rodrigues-Costa et al., 2018). Remarkably, we detected a ST barely reported in studies around the world, the ST317, belonging to the still small CC317. CC317 was identified in 26.2% of all isolates (belonging to 11 different pulsotypes) in this study, representing the most frequent ST in BSI over the years 2008 to 2010. According to MLST database, the first ST317 isolate was detected in 2009 from an unknown sample in Rio de Janeiro, Brazil (https://pubmlst.org/abaumannii/). The only other strain deposited at the MLST database was detected in the State of São Paulo, isolated from upper respiratory tract secretion in 2011 (Camargo et al., 2016), likely indicating a limited spread of this clone, only in Brazil. Although very prevalent among the isolates studied herein, CC317 seemed to be replaced by other clones (ST79 and ST730), which belong to CC79. Conversely, analysis of our data indicate that CC317 presented less pronounced antimicrobial resistance to sulfamethoxazole-trimethoprim, tigecycline, and ampicillin-sulbactam, indicating that, at least in parts, antimicrobial resistance can drive changes in prevalence of clones in specific settings under selective pressure.

In the latest years of the study, however, the emergence of strains belonging to CC1 likely indicate that another epidemiologic shift has occurred. Changes in clonal structure of *Acinetobacter* strains were reported in other hospitals (Park et al., 2012; Villalón et al., 2015), but the reasons to explain this shift remain to be totally understood. In our study, a remarkable diversity of pulsotypes identified into each of these clonal complexes suggests that selective pressure, instead of a strict clone spread, plays a more decisive role in the emergence of CRAB.

## Conclusion

In summary, we observed a change in the prevalence of CRAB clones in a single hospital, despite the persistence of OXA-23-producing isolates with MDR or XDR phenotypes, possibly driven by antimicrobial resistance and selective pressure. Longitudinal studies, as the present, provide this type of temporal observation, making possible to track the dispersion dynamics of successful clones associated with the persistence of well-stablished resistance phenotypes in *Acinetobacter baumannii* and to propose measures to contain its dissemination.

## Ethics statement

This study was submitted and approved by the Adolfo Lutz Institute Ethics Committee under the register number CAAE 49985115.5.0000.0059.

## Author contributions

LT and CC conceived and designed the study, analyzed the data, and wrote the paper. LT, FV, and WS performed the experiments. MT-C, CC, TR, ALM, AF, ACM, and TS contributed reagents, materials, analysis tools.

### Conflict of interest statement

The authors declare that the research was conducted in the absence of any commercial or financial relationships that could be construed as a potential conflict of interest.
